# Stereotactic body radiation therapy (SBRT) in patients with hepatocellular carcinoma and oligometastatic liver disease

**DOI:** 10.1186/s13014-018-1048-4

**Published:** 2018-05-29

**Authors:** Sabine Gerum, Christian Heinz, Claus Belka, Franziska Walter, Philipp Paprottka, Enrico N. De Toni, Falk Roeder

**Affiliations:** 1 Department of Radiation Oncology, University Hospital LMU Munich, Marchioninistr. 15, 81377 Munich, Germany; 2 Department of Radiology, University Hospital LMU Munich, Marchioninistr. 15, 81377 Munich, Germany; 3 Department of Internal Medicine, University Hospital LMU Munich, |Marchioninistr. 15, 81377 Munich, Germany; 4 CCU Molecular Radiation Oncology, German Cancer Research Center, Heidelberg, Germany

**Keywords:** Liver, HCC, Oligometastatic, SBRT

## Abstract

**Background:**

To report our experience with SBRT in primary and secondary liver tumors.

**Methods:**

We retrospectively analysed 55 patients (70 lesions) with a median follow-up of 10 months (range 1–57) treated from 2011 to 2016. All patients had not been eligible for other local treatment options. Median age was 64 years and 64% were male. 27 patients (36 lesions) suffered from hepatocellular carcinoma (HCC, Child A:78%, Child B:18%, Child C:4%), 28 patients (34 lesions) had oligometastatic liver disease (MD). Treatment planning was based on 4D-CT usually after placement of fiducials. Dose and fractionation varied depending on localization and size, most commonly 3 × 12.5 Gy (prescribed to the surrounding 65%-isodose) in 56% and 5x8Gy (80% isodose) in 20% of the treated lesions.

**Results:**

Local recurrence was observed in 7 patients (13%) and 8 lesions (11%), resulting in estimated 1- and 2-year local control rates (LC) of 91 and 74%. Estimated 1- and 2-year rates of Freedom from hepatic failure (FFHF) were 42 and 28%. Number of lesions was predictive for LC and FFHF in the entire cohort. Estimated 1- and 2-year overall survival (OS) was 76 and 57%. OS was significantly affected by number of treated lesions and performance status. In the HCC subgroup, pretreatment liver function and gender were also predictive for OS. Maximum acute non-hepatic toxicity was grade 1 in 16% and grade 2 in 10% of the patients. Three HCC patients (11%) developed marked deterioration of liver function (grade 3/4).

**Conclusions:**

SBRT resulted in high local control and acceptable survival rates in patients with HCC or MD not amendable to other locally-ablative treatment options with limited toxicity. Care should be taken in HCC patients with Child B cirrhosis.

## Background

Primary and metastatic liver tumors are among the most common malignancies and tumour-related causes of death worldwide [1, 2]. Treatment paradigms have changed dramatically in the last decades in favor of local treatments in primary liver cancers and oligometastatic (especially liver-confined) disease because of the evolving evidence for possible cure or at least long-term survival [3, 4]. Treatment options range from liver transplantation (for HCC) or extended surgical resections (for MD) to less invasive techniques like radiofrequency ablation (RFA), transarterial chemoembolization (TACE) or selective internal radiotherapy (SIRT). However, the potential benefit of such treatment options needs to be weighed against the possibility that local treatment results in impairment of liver function or liver failure, especially in the presence of an underlying liver disease, which is the background upon most primary malignancies arise [4]. Moreover, any of the mentioned treatments has its limitations. For example surgery is often limited by comorbidities or poor liver function [4] while lesions directly adjacent to major vessels or bile ducts are not well suited for RFA [5] and patients with portal vein thrombosis are not eligible for TACE [6].

Stereotactic body radiation therapy (SBRT) is a highly conformal technique of percutaneous radiation therapy delivered in a small number of large fractions [7]. It sufficiently spares dose to adjacent organs at risk due to its sharp dose fall-off outside the target, while adequate tumor control is maintained due to the enhanced biological effectivity of the large single doses. Stereotactic radiation approaches have been already successfully introduced into the treatment of primary and secondary brain and lung tumors and have shown to result in low toxicity and at least comparable outcome with regard to surgery [7–10]. Several groups have shown that SBRT can also be effectively employed with acceptable toxicity for the treatment of liver malignancies [11]. However, no randomized trials comparing SBRT to other local treatment options have been conducted so far, and only scarce prospective data on the employment of SBRT in the treatment of liver lesions are available. Moreover, no generally accepted criteria for patient selection or a generally accepted dose and fractionation concept exists. Therefore we report our experience with SBRT for primary and secondary liver tumors.

## Methods

**Fig. 1:**
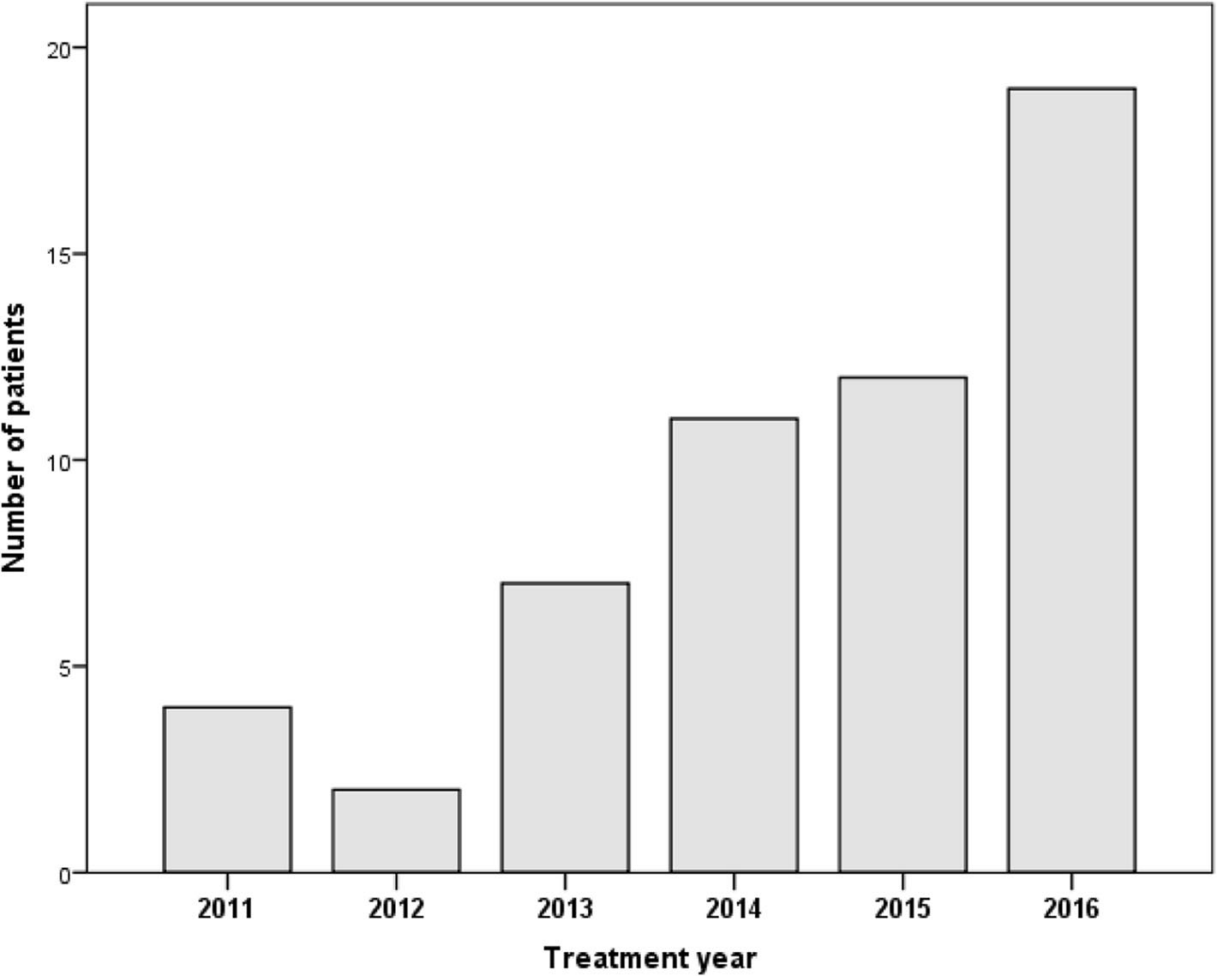
Number of treatments per year

In our institution, SBRT has been used for the treatment of malignant liver lesions for the first time in 2011. Since then, an increasing number of patients have been treated each year (fig. 1). For the current analysis, we retrospectively selected and analyzed all patients affected by HCC or MD who underwent SBRT to 1–3 liver lesions. Indication for SBRT was seen in patients not eligible for other local treatment options according to multidisciplinary evaluation. Pretreatment investigations included MRI and/or contrast-enhanced biphasic liver CT, liver function tests for HCC patients and additional CT/PET-CT staging for MD patients.

### Patient characteristics

The entire cohort consisted of 55 patients with 70 treated lesions. Median age was 64 years (31–83), 64% were male and the median Karnofsky performance score (KPS) was 90% (60–100%). 28 patients showed MD in whom 34 lesions were treated. Most of them suffered from colorectal cancer (32%) followed by NSCLC (14%) and soft-tissue sarcomas (14%). 27 patients showed HCC in whom 36 lesions were treated. Underlying liver cirrhosis was present in all HCC patients, caused by viral hepatitis (B and C) in 10 patients (37%), alcohol consumption in 5 (19%), autoimmune hepatitis in 1 (4%) and by unknown reason in 11 patients (41%). Liver function was moderately restricted in the majority of them (Child A: 21[78%], Child B: 5[18%], Child C: 1[4%]), Table 1. The patient with Child C cirrhosis presented with good performance score and was listed for liver transplantation, therefore our interdisciplinary tumorboard opted for SBRT as bridging.

**Table 1:** patient and treatment characteristics

	entire cohort	HCC	MD
number of patients	55	27	28
number of lesions	70	36	34
single/multiple lesions	40/15	18/9	22/6
gender (male/female)	35/20	19/8	16/12
age (median/range in years)	64/31–83	63/46–83	67/31–80
KPS (median/range in %)	90/60–100	90/60–100	95/70–100
histology (CRC/LC/BC/S/O)	–	–	10/4/4/2/9
Child-Pugh stage (A/B/C)	–	21/5/1	–
fiducials (n/%)	42/76	18/67	24/86
abdominal compression (n/%)	26/47	14/52	12/43
GTV per patient (median/range in ccm)	14.6/0.4–204	15.5/1.2–204	13.25/0.4–98.2
GTV per lesion (median/range in ccm)	6.6/0.2–204	7.5/0.9–204	6.2/0.2–98.2
PTV per patient (median/range in ccm)	75.5/17.7–511.6	92.7/17.7–511.6	61.1/19.3–314
PTV per lesion (median/range in ccm)	59/15–512	61/15–512	51/15–314
dose concept (3 × 12.5/5 × 8/other)	28/11/16	16/6/5	12/5/9
BED max (median/range)	168.1/60.3–190	168.2/60.3–168.2	118/80.4–190
actual treatment (SBRT/TACE+SBRT)	–	12/15	–

*HCC*: hepatocellular carcinoma, *MD*: metastatic disease, *KPS*: Karnofsky performance score, *CRC*: colorectal cancer, *LC*: lung cancer, *BC*: breast cancer, *S*: sarcoma, *o*: other, *GTV*: gross tumor volume [cubic centimeters], *PTV*: planning target volume [cubic centimeters], *BED*: biologically equivalent dose, *SBRT*: stereotactic body radiotherapy, *TACE*: transarterial chemoembolization, all doses in [Gy]

**Table 2:** univariate analysis for LC, FFHF and OS

	LC		FFHF		OS	
	1-year rate	*p* value	1-year rate	*p* value	1-year rate	*p* value
gender						
male	88%	0.279	44%	0.679	83%	0.055
female	94%		41%		67%	
age						
≤ median (64 yrs)	92%	0.265	40%	0.687	77%	0.76
> median	90%		43%		75%	
histology						
HCC	92%	0.492	41%	0.895	68%	0.701
MD	89%		40%		84%	
KPS						
≤70	100%	0.425	42%	0.579	50%	**0.028**
> 70	90%		42%		79%	
GTV						
≤ median (14.6 ccm)	100%	0.535	46%	0.794	80%	0.416
> median	88%		44%		77%	
BED						
≤ 100	87%	0.651	38%	0.419	75%	0.806
> 100	94%		48%		77%	
lesions						
single	97%	**0.011**	49%	**0.047**	80%	**< 0.001**
multiple	67%		19%		40%	

*LC*: Local control, *FFHF*: Freedom from hepatic failure, *OS*: Overall survival, *yrs.*: years, *HCC*: hepatocellular carcinoma, *MD:* metastastic disease*, KPS*: Karnofsky performance score, *GTV*: Gross tumor volume (measured per patient on free-breathing CT), ccm: cubic centimeters, *BED:* biological equivalent dose

### Treatment characteristics

Treatment in HCC patients consisted of SBRT alone in 12 patients while 15 patients received selective TACE to the same lesions upfront to SBRT (within 6 weeks). Five patients received additional RFA treatments to different lesions prior to SBRT (within 6 weeks). 23/27 patients had a median of 2 (range 1–8) previous local treatments (surgery, RFA, TACE or SIRT). Treatment of metastatic patients consisted of SBRT alone in 25 patients or SBRT combined with surgery or RFA to different lesions (within 6 weeks) in 3 patients. Primary tumor was controlled in all patients with MD at the time of SBRT. Additive systemic therapy within three months from SBRT was given to 1 patient with HCC and 8 patients with MD.

### SBRT

Prior to SBRT, 42 patients received CT-guided implantation of 1–3 fiducials (Visicoil™, IBA dosimetry or MPB™, MPB Scherer Medizinprodukte) per lesion unless enhancement of lipiodol in patients with prior TACE (*n* = 8) or the presence of surgical clips (*n* = 5) were deemed sufficient to guide the procedure. Patients were immobilized using a vacuum pillow in combination with an alpha-cradle. Abdominal compression was used since 2014 (*n* = 26, 47%). Treatment planning was based on contrast-enhanced 4D-CT. Gross tumor volume (GTV) was contoured as the visible tumor on the free-breathing CT and on all respiratory phases of the 4D-CT supplemented by information from MRI if available. An internal target volume (ITV) was constructed and enlarged by an isotropic margin of 6 mm to obtain the planning target volume (PTV). Dose was prescribed to the PTV surrounding isodose in all patients. Prescription isodose, single dose and number of fractions depended on size and location of the lesions. Number of lesions did not influence prescription dose in general. The most common schemes were 3 × 12.5 Gy (65%-isodose) in 56% and 5x8Gy (80%-isodose) in 20% of the treated lesions delivered every other day. Implanted fiducials or lipiodol enhancement were contoured accordingly to receive a fiducial or lipiodol ITV, which was used for daily patient set-up. Treatment was performed using daily CBCT image-guidance.

### Statistical and legal considerations

Regular follow-up examinations (including physical examination, laboratory tests (liver function and tumor marker tests), MRI/CT of the liver) took place at our department or the departments of gastroenterology/oncology every three months for one year, every 6 months for the second and annually thereafter. Toxicity was scored retrospectively according to CTCAE v4.03. Because of the retrospective nature not all patients had received exactly the same laboratory tests. Therefore changes in hepatic laboratory tests without symptoms were not counted as toxicity. Marked deterioration of liver function was defined as change in CHILD-Pugh class from A to B or B to C in HCC patients or symptomatic changes in liver function in MD patients. Biological effective dose (BED) of the maximum PTV dose was calculated according to the LQ formalism: BED = n*d*(1 + d/{α/β}) with n being the number of fractions, d the daily single fraction dose and alpha-beta for tumor tissue of 10 Gy. Local control (LC) was defined as absence of tumor progression in the region of the treated lesion. Freedom from hepatic failure (FFHF) was defined as absence of tumor progression in the liver. All time-to-event data was calculated from the first day of SBRT using the Kaplan-Meier method. All endpoints and subgroup analyses are reported referring to patients (not lesions) if not otherwise specified. Differences in subgroups were assessed by the logrank test for univariate analysis. Due to the low number of events multivariate analysis was not performed. The pearsons test was used for evaluation of possible correlations between parameters. A *p*-value of < 0.05 was defined as statistically significant. The analysis was in accordance to the declaration of Helsinki in its latest version and was approved by our independent Ethics committee.

## Results

Median follow-up in all patients was 10 months (1–57) and 13 months in survivors. Since implementation of the technique at our center in 2011 we have seen a continuous increase in patient numbers per year resulting in 56% of the included patients treated in 2015/16 (fig. 1). Median GTV (measured on free-breathing CT) per patient was 14.6 ccm (0.4–204) and 6.6 ccm per lesion (0.2–204) and median PTV was 75.5 ccm (17.7–511.6) per patient and 59 ccm (15–512) per lesion.

### LC

**Fig. 2:**
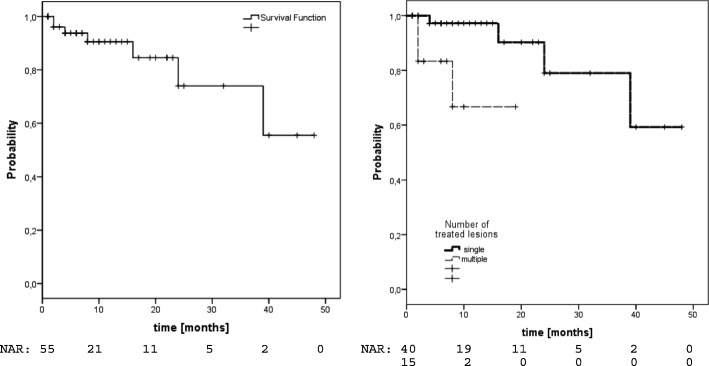
Local control left: entire cohort, right: according to number of treated lesions

Local recurrence was observed in 7/55 patients (13%) translating into estimated 1- and 2-year LC-rates of 91 and 74% (fig. 2). Median time to local failure in these 7 patients was 8 months (2–39). In univariate analysis, only the number of lesions was predictive for LC (1-year-LC single 97% vs. multiple 67%,*p* = 0.011, Fig. 2, Table 2). In the HCC group we observed estimated 1- and 2-year LC-rates of 92%. The number of lesions was the only factor with significant impact on LC (1-year-LC single 100% vs. multiple 71%,*p* = 0.024). No significant difference was observed comparing patients with TACE+SBRT versus SBRT alone. In the MD group, we observed estimated 1- and 2-year LC-rates of 89 and 64%. Improved LC was significantly associated with treatment of a single lesion (1-year-LC 95% vs. 0%,*p* = 0.027), BED>150Gy (1-year-LC 100% vs. 82%,*p* = 0.036) and female gender (1-year-LC 100% vs. 78%,*p* = 0.039). We further analyzed possible associations between the number of lesions and dose parameters (BED maximum, prescription dose) but did not find any significant correlations (data not shown).

If LC was analysed per lesion, we observed local failures in 8/70 lesions (11%), translating into 1- and 2-year LC-rates of 91 and 74%. No factors with significant impact on LC could be identified for the entire cohort or the HCC subgroup. However, regarding the MD subgroup, we observed a significant association of LC with GTV volume (1-year LC GTV ≤6.25ccm 100% vs. 80% with GTV > 6.25ccm;*p* = 0.041).

### FFHF

**Fig. 3:**
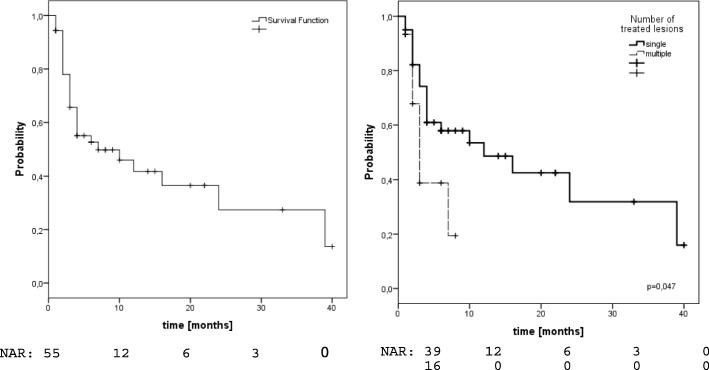
Freedom from hepatic failure left: entire cohort, right: according to number of treated lesions

29/55 patients (53%) showed hepatic failure of whom only 1 had an isolated local failure, while 22 showed isolated failures outside the treated volume and 6 had combined failures. Estimated 1- and 2-year FFHF-rates were 42 and 28% (fig. 3). Again, only the number of lesions (1-yr-FFHF single 49% vs. 19% multiple,*p* = 0.047) was predictive for FFHF (Fig. 3, Table 2). In the HCC subgroup, we observed estimated 1- and 2-year FFHF-rates of 41%. FFHF was significantly affected by the number of lesions (1-year-FFHF single 53% vs 15% in multiple,*p* = 0.01). In the MD subgroup we found estimated 1- and 2-year FFHF-rates of 40 and 25%. FFHF was significantly associated with performance status (1-year-FFHF KPS ≤ 70 0% vs. 43% with KPS > 70,*p* = 0.006).

### OS

**Fig. 4:**
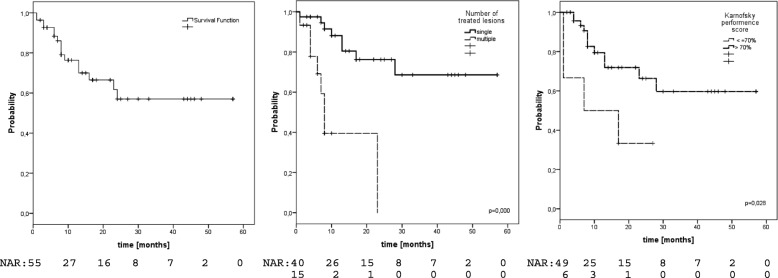
Overall Survival left: entire cohort, middle: according to number of treated lesions, right: according to performance status

16 patients (29%) have died, translating into estimated 1- and 2-year OS-rates of 76 and 57% (Fig. 4). Prognostic factors regarding OS were number of treated lesions (1-yr-OS single 88% vs 40% in multiple,*p* < 0.001, Fig. 4) and performance status (1-yr-OS KPS > 70 79% vs 50% KPS ≤ 70,*p* = 0.028, Fig. 4), see Table 2. A trend was also observed for gender (1-yr-OS male 83% vs 67% female, *p* = 0.055). In the HCC subgroup we observed estimated 1- and 2-year OS-rates of 68 and 57%. OS was significantly associated with number of treated lesions (1-year-OS single 84% vs 39% multiple,p < 0.001), pretreatment liver function (1-year-OS Child-Pugh A 76% vs. Child-Pugh B 28%,*p* = 0.036) and gender (1-year-OS male 83% vs. female 43%,*p* = 0.049). In the MD subgroup we observed estimated 1- and 2-year OS-rates of 84 and 67%. OS was significantly associated with performance status (1-year-OS KPS > 70 90% vs. 33% KPS ≤ 70,p < 0.001) and number of treated lesions (1-year-OS single 90% vs. 42% multiple,p = 0.036).

### Toxicity

CT-guided fiducial placement was feasible without any complications. SBRT treatment could be performed as planned in all except one patient (2%) who prematurely finished radiation treatment due to humerus fracture. Maximum acute toxicity was grade 1 in 9 patients (16%), grade 2 in 6 (10%), grade 3 in 2 (4%) and grade 4 in 1 patient (2%), see Table 3. The most frequent side effects were fatigue and gastrointestinal symptoms like mild nausea or diarrhea. Three patients with HCC (11%) developed marked deterioration of liver function without disease progression. Two had a decline in Child-Pugh stage from A to B. One patient with Child-Pugh C cirrhosis developed hepatic failure (grade 4) shortly after SBRT which was successfully treated by liver transplantation.

**Table 3:** Acute toxicity

	CTCAE °I	CTCAE °II	CTCAE °III	CTCAE °IV
	n (%)			
nausea (n/%)	4 (7)	4 (7)		
fatigue	3 (5)			
pneumonitis	1 (2)			
tachyarrhythmia	1 (2)			
cholangitis		1 (2)		
diarrhea		1 (2)		
severe deterioration of liver function	n.a.	n.a.	2 (4)	1 (2)

*n.a*.: not applicable, *CTCAE:* common toxicity criteria for adverse events Version 4.03

## Discussion

### Outcome with SBRT

Regarding LC, we observed 1- and 2-year-rates of 91 and 74% for the entire cohort. LC seemed slightly improved in the HCC group with 1- and 2-year-rates of 92% compared to the MD group with 1- and 2-year-rates of 89 and 64%, although this difference did not reach statistical significance. These results are in line with other series including both primary and secondary liver tumors treated with similar concepts. For example Mendez-Romero et al. [12] described their results of a phase I-II trial including 25 patients with HCC or MD treated mainly with a 3 × 12.5Gy concept and found 1- and 2-year LC-rates of 91 and 82%. If HCC and MD are considered separately, our results are still in the published range of 75–100% 1-year-LC in HCC [4] and 71–94% 1-year-LC in MD patients [13] reported in recent major series (Table 4).

**Table 4:** Selected SBRT series in patients with primary and secondary liver tumors

Author, Year Study-Type	n (patients) n (lesions)	n (primary LT) n (metastases)	GTV volume (median/range)^a^	1 yr-LC (%) (HCC/MD)	1 yr-OS (%) (HCC/MD)	Toxicity Gr3+ (%)
Mendez-Romero et al. 2006 Phase I/II	25 45	HCC: 11 MD: 34	3.5 (0.5–7.2)	94 (75/100)	82 (75/85)	12.5
Goodman et al. 2010 Phase I	26 40	HCC/CCC: 7 MD: 19	32.6 (0.8–146.6)	77	62	0
Own data	55 70	HCC: 27 MD: 28	6.6 (0.2–204)	91 (92/89)	76 (68/84)	5^b^
Bujold et al. 2013 Phase I/II	102	HCC only	7.2 (1.4–23.1)	87	55	30
Lasley et al. 2015 Phase I/II	59 59	HCC only	33.6 (2.2–107.3)	91 (Child A) 82 (Child B)	94 (Child A) 57 (Child B)	11 (Child A) 38 (Child B)
Own data	27 36	HCC only	7.5 (0.9–204)	92	68	11^b^
Scorsetti et al. 2015 Phase I/II	42 52	MD only	3.5 (1.1–5.4)	91 (2 yr)	65 (2 yr)	0
Andratschke et al. 2015 Retro, pooled	74 91	MD only	n.r.	75	77	0
Own data	28 34	MD only	6.2 (0.2–98.2)	89	84	0

*n:* number, *LT*: liver tumors, *GTV*: gross tumor volume per lesion, *yr.*: year, *LC*: local control, *OS*: overall survival, *HCC*: hepatocellular carcinoma, *MD*: metastatic disease, Gr3+: grade 3+, ^a^ in cubic centimeters, ^b^: deterioration of liver function, no other grade 3+ toxicity, n.r.: not reported

With regard to OS, we observed 1- and 2-year-rates of 76 and 57% for the entire cohort. OS seemed to be slightly better in the MD group with 1- and 2-year OS-rates of 84 and 67% compared to the HCC group with 1- and 2-year OS-rates of 68 and 57%, although this difference did not reach statistical significance. Mendez-Romero et al. [12] described similar outcomes with 1- and 2-year OS-rates of 82 and 54% for the whole group, 85 and 62% for patients with MD and 75 and 40% for patients with HCC. Moreover, our results are in the range of published results focusing on primary or secondary liver tumors with 1- and 2-year OS-rates of 62–85% and 38–70% in metastatic patients [3] and 55–100 and 53%–69% in HCC patients [4].

In contrast to the encouraging rates for LC and OS, we observed rather poor 1- and 2-year FFHF-rates of 42 and 28% for the entire cohort, which were mainly driven by intrahepatic outfield failures indicating a high risk for the development of new lesions in these heavily pretreated patients. This pattern was seen in patients with HCC (1- and 2-year FFHF 42%) as well as in patients with MD (1- and 2-year FFHF 40 and 25%) although more pronounced in the latter group. Similar results have been reported by others for both entities. For example Yoon et al. [14] found a crude rate of 63% intrahepatic outfield failures and 71% hepatic failures in total, translating into a 1-year hepatic-failure-free-survival-rate of 52% in their cohort of 92 patients with HCC treated with SBRT. Chang et al. [15] reported a crude rate of 68% intrahepatic outfield failures in their study on 65 patients with colorectal liver metastases.

### Prognostic factors

We analyzed our entire cohort and both subgroups (HCC and MD) with regard to possible prognostic factors for each endpoint. Regarding LC, lesion size [16–18] and dose of SBRT [5, 15, 16, 18–20] have been the most consistently reported prognostic factors for both groups. Although a variety of definitions and tresholds have been used and some studies did not found any associations at all [19–22]. We could not confirm a statistically significant association of GTV volume or BED with LC regarding the entire cohort and the subgroup of HCC patients. However, we observed a significantly reduced LC-rate with lower BED and larger GTV volume in the subgroup of patients with MD, thus emphasizing the findings of others suggesting a relationship between LC and lesion size and/or dose at least in metastatic patients. Interestingly, the number of lesions was the main factor in our study with a significant impact on LC, however this might be simply due to statistical reasons as patients with more lesions obviously have a high probability to fail at least in one.

Regarding OS, an even larger variety of factors with possible impact have been described in the literature, including gender [23], number of lesions [22], lesion diameter [15, 23], GTV volume [16, 17] and dose [24] for HCC and/or MD as well as Child-Pugh stage for HCC [17, 24] and histology for MD [16, 22]. However, the prognostic value of those factors is far from being consistently evident as negative or even opposing results have been reported also for any of the mentioned factors [15, 16, 20, 21, 23, 25]. In our study, we identified the number of lesions and performance status as factors associated with OS for the entire cohort. In the HCC subgroup Child-Pugh stage and gender were additional factors with significant impact. Although conflicting data exists, it seems reasonable to assume that patients with multiple lesions are at higher risk for the development of consecutive lesions with consequently reduced survival, especially in a patient group like ours with limited salvage options. This is further supported by our data with regard to the clearly reduced FFHF in patients with multiple lesions. The same assumption seems true for performance status which has been shown in many other oncological situations to be a key factor in predicting outcome [26, 27]. Regarding the HCC subgroup, it has been shown that OS is clearly associated with Child-Pugh stage [28]. It therefore seems no surprise that OS of patients with HCC lesions (which developed on the basis of advanced liver cirrhosis) is affected by the severity of the underlying cirrhosis. In contrast, the observed negative influence of female gender on OS of HCC patients is difficult to explain. Huertas et al. [23] reported a similar result in their HCC series even according to multivariate analysis, however Yamashita et al. [25] found the opposite association leaving this question unanswered.

### Toxicity

Given the high rate of outfield failures with the need for salvage treatments, toxicity and preservation of liver function are of important value in the decision process for locally-ablative treatments. With our approach, we observed high treatment compliance with predominantly mild toxicities mainly including fatigue and nausea. Three patients with HCC (11%) developed marked deterioration of liver function. Similar results have been published in most other series. For patients treated for MD, acute and late grade3+ toxicities are reported in the range of 0–16% and 0–5% [3]. Similar to our results, Andratschke et al. [16] reported mild side effects mainly consisting of fatigue and nausea but no grade3+ reactions in their series of 74 patients treated with comparable dose and fractionation concepts. In patients with HCC higher complications rates have been observed. Acute grade3+ toxicities ranged from 5 to 37% including up to 7% deaths [4], mainly in Child-Pugh B patients. Decline in Child-Pugh class has also been reported in 13–29% [12, 20, 24], although some authors described a marked recovery over time [20]. Our toxicity rate seem to compare favorably with those rates, however some of these studies included larger lesions resulting in more dose to normal liver tissue and a higher percentage of Child-Pugh B patients. Both factors have been shown to be associated with toxicity [24, 29]. For example Andolino et al. [24] described progressive liver dysfunction in 4/8 patients with Child-Pugh≥8 of whom 2 could be salvaged by transplant but 2 died. They concluded to further treat Child-Pugh B patients only if listed for transplantation. Lasley et al. [29] observed grade 3/4 liver toxicity of 11% in patients with Child-Pugh A compared to 38% in Child-Pugh B patients and further excluded patients with Child-Pugh≥8 from treatment. Finally, Culleton et al. [30] analyzed specifically patients with Child-Pugh B/C and described a decline of ≥2 points in 63% at 3 months. Therefore SBRT treatment should be used with caution or restricted in dose in patients with already restricted pretreatment liver function (Child Pugh B) while SBRT seems to be generally well tolerated in patients with Child Pugh A or in patients with MD. Moreover, a recent systematic review including 5 studies with 392 patients suffering from primary and secondary liver tumors treated with SBRT demonstrated well-preserved post-treatment quality-of-life at least comparable or even favorable compared to other surgical or non-surgical approaches [1].

### Limitations

Clearly our analysis has some limitations, namely its retrospective nature, the small sample size, the mixed cohort and the rather short follow-up. However, in the absence of prospective randomized trials and only a limited number of prospective studies reported in the literature our experience may help clinicians and researchers to guide their further decisions.

## Conclusions

SBRT resulted in high LC and acceptable survival rates in patients with HCC or MD not amendable to other locally-ablative treatments. However, especially patients with multiple lesions are at high risk for intrahepatic outfield failures indicating a possible need for additional therapies. OS was predicted by number of lesions and performance status as well as pretreatment liver function in HCC patients. Toxicity was generally mild. High grade toxicity was restricted to patients with HCC suffering from underlying cirrhosis Child-Pugh class B indicating the need for special attention in those patients.

## Abbreviations

4D-CT: Four-dimensional computed tomography
BED: Biologically effective dose
CBCT: Cone beam computed tomography
ccm: Cubic centimeters
CT: Computed tomography
CTCAE: Common toxicity criteria for adverse events
FFHF: Freedom from hepatic failure
GTV: Gross tumor volume
Gy: Gray
HCC: Hepatocellular carcinoma
ITV: Internal target volume
KPS: Karnofsky performance score
LC: local control
MD: Oligometastatic disease
MRI: Magnetic resonance imaging
OS: Overall survival
PET-CT: Positron-emission-tomography with computed tomography
PTV: Planning target volume
RFA: Radiofrequency ablation
RILD: Radiation induced liver disease
SBRT: Stereotactic body radiation therapy
SIRT: Selective internal radiotherapy
TACE: Transarterial chemoembolization
